# Pancreatic cancer-educated macrophages protect cancer cells from complement-dependent cytotoxicity by up-regulation of CD59

**DOI:** 10.1038/s41419-019-2065-4

**Published:** 2019-11-04

**Authors:** Ronghua Zhang, Qiaofei Liu, Junya Peng, Mengyi Wang, Xiang Gao, Quan Liao, Yupei Zhao

**Affiliations:** 1 Department of General Surgery, Peking Union Medical College Hospital, Peking Union Medical College & Chinese Academy of Medical Sciences, Beijing, 100730 China; 2 Department of Center Lab, Peking Union Medical College Hospital, Peking Union Medical College & Chinese Academy of Medical Sciences, Beijing, 100730 China

**Keywords:** Cell death and immune response, Immunization

## Abstract

Tumor-associated macrophages (TAMs) are versatile immune cells that promote a variety of malignant behaviors of pancreatic cancer. CD59 is a GPI-anchored membrane protein that prevents complement activation by inhibiting the formation of the membrane attack complex, which may protect cancer cells from complement-dependent cytotoxicity (CDC). The interactions between CD59, TAMs and pancreatic cancer remain largely unknown. A tissue microarray of pancreatic cancer patients was used to evaluate the interrelationship of CD59 and TAMs and their survival impacts were analyzed. In a coculture system, THP-1 cells were used as a model to study the function of TAMs and the roles of pancreatic cancer-educated macrophages in regulating the expression of CD59 in pancreatic cancer cells were demonstrated by real-time PCR, western blot and immunofluorescence staining. The effects of macrophages on regulating CDC in pancreatic cancer cells were demonstrated by an in vitro study. To explore the potential mechanisms, RNA sequencing of pancreatic cancer cells with or without co-culture of THP-1 macrophages was performed, and the results showed that the IL-6R/STAT3 signaling pathway might participate in the regulation, which was further demonstrated by target-siRNA transfection, antibody neutralization and STAT3 inhibitors. Our data revealed that the infiltration of TAMs and the expression of CD59 of pancreatic cancer were paralleled, and higher infiltration of TAMs and higher expression of CD59 predicted worse survival of pancreatic cancer patients. Pancreatic cancer-educated macrophages could protect cancer cells from CDC by up-regulating CD59 via the IL-6R/STAT3 signaling pathway. These findings uncovered the novel mechanisms between TAMs and CD59, and contribute to providing a new promising target for the immunotherapy of pancreatic cancer.

## Introduction

Pancreatic cancer has a poor prognosis and a rising incidence^1,2^. Pancreatic ductal adenocarcinoma (PDAC), is the most common type and accounts for 90% of all pancreatic cancer cases^3^. Immune checkpoint inhibitors, including therapies against cytotoxic T-lymphocyte-associated protein 4 (CTLA-4) and programmed cell death protein 1 (PD-1), have played limited roles in treating pancreatic cancer^4,5^. Therefore, new immunotherapeutic strategies for pancreatic cancer are urgently needed.

The complement system is a crucial part of the immune system and protects the host from pathogenic microorganisms and damaged cells^6^. The benefit of mAb-based immunotherapies, such as rituximab and ofatumumab, is attributed partially to their ability to evoke complement-dependent cytotoxicity (CDC) that eliminates tumor cells^6,7^. CD59 is a glycosylphosphatidylinositol (GPI)-anchored membrane protein that regulates complement activation by preventing C9 from polymerizing and forming the membrane attack complex (MAC)^8^. CD59 was reported to be highly expressed in clinical patients with pancreatic cancer^9,10^. However, the role of CD59 in the prognosis of pancreatic cancer has not been reported. Tumor-associated macrophages (TAM) have been demonstrated to play an important role in the processes of tumor carcinogenesis, including the escape of cancer cells from the tumor into the circulation and the suppression of antitumor immune functions and drug resistance^11^. Studies of the immunosuppressive functions of TAMs have mainly focused on tumor-promoting cytokines and their suppressive effects on T cell function. The effects of TAMs on the functions of the complement system have rarely been reported.

In this study, the interactions and mechanisms of TAMs, CD59 and pancreatic cancer were studied, to uncover new immunotherapeutic targets for pancreatic cancer.

## Material and methods

### PDAC sample collection and tissue microarray construction

PDAC tumor tissues and corresponding adjacent nontumor tissues after radical resection with an R0 tumoral margin were collected from 74 patients aged 34–85 using the following inclusion criteria: (1) all of the patients had complete clinico-pathological information and a follow-up visit; (2) the tumor tissues were histologically proven to be ductal adenocarcinoma; (3) both the paired tumor and nontumor tissues were obtained; and (4) all of the patients did not undergo neoadjuvant chemotherapy. Tumor staging was based on the 8th edition of the TNM system designed by the American Joint Committee on Cancer (AJCC). All samples were used to construct tissue microarrays. None of the patients died before the follow-up visit. While 52 of 74 cases died, the remaining 22 cases were still alive until the end of the follow-up period, which ranged from 5 to 87 months after resection. This study was approved by the Ethics Committee of Peking Union Medical College Hospital, and all the patients signed the informed consent form. We used SurvExpress^12^ to evaluate the relationship between CD59 expression and cancer risks in the TCGA Pancreatic Carcinoma Dataset. Through the SurvExpress program, the CD59 expression levels of patients in the TCGA dataset, which were divided into “Low Risk” and “High Risk” groups according to the prognostic index, were analyzed (http://bioinformatica.mty.itesm.mx:8080/Biomatec/SurvivaX.jsp). We also explored the correlation between CD59 expression and overall survival (OS) in pancreatic cancer patients by the Kaplan-Meier plotter (http://kmplot.com), a widely used online database^13^.

### Immunohistochemistry (IHC) assay and evaluation

The pancreatic cancer tissue microarray above was used to evaluate the expression of CD59 and CD163 in tumor and peritumor tissue. The sections were then deparaffinized in xylene, rehydrated according to standard histopathological procedures and stained with hematoxylin and eosin. The slides were then incubated with 1:200 dilutions of CD59 antibody (HPA026494, Sigma-Aldrich) and CD163 antibody (ab182422, Abcam). The stained tissues were scored by two pathologists blinded to the patient information. The expression level of CD59 was evaluated by the H-score^14^, a widely used evaluation criterion for immunostaining that involves multiplying a proportion score and an intensity score^15–17^. The H-score value with the largest Youden’s Index for each variable within the receiver operating characteristic (ROC) curve was selected as the cut-off. The specimens with an H-score above or equal to the cut-off value were defined as those with high expression of CD59, whereas the others were regarded as having low CD59 expression. CD163 is a commonly accepted marker for tumor-promoting TAMs^11^. The CD163-positive macrophages in the stained sections were counted using ImageScope software, and the density was estimated (per square millimeter) at a higher magnification (×200). The cut-off value was selected by the ROC curve as mentioned above.

### Cell culture and treatment

Seven pancreatic cancer cell lines, namely, BxPC-3, Mia PaCa-2, T3M4, PANC-1, AsPC-1, Su86.86, and CFPAC-1, were maintained in a humidified incubator with 5% CO_2_ at 37 °C in RPMI-1640 medium or Dulbecco’s modified Eagle’s medium (DMEM, HyClone, Thermo Fisher Scientific Inc., Waltham, MA, USA) containing 10% fetal bovine serum (FBS, HyClone) and 1% penicillin and streptomycin.

To determine whether CD59 expression was related to the cross-talk between cancer cells and TAMs, we created a transwell coculture system. Human monocyte THP-1 cells were used as a model to study the function of TAMs according to previously reported protocols^18^ and THP-1 cells (2.5 × 10^5^/mL) were induced into naive macrophages (Mφ group) using phorbol-12-myristate-13-acetate (PMA; 100 ng/mL, Sigma-Aldrich, USA) for 24 h. Pancreatic cancer cells (2.0 × 10^5^/mL) were seeded into 6-well plates (Corning, NY, USA). After 24 h, naive Mφ cells were seeded into the upper compartment of a transwell with a 0.4-μm pore size (Corning, NY, USA) with DMEM or RPMI-1640 medium supplemented with 10% FBS. Then, the upper and lower compartments were combined and cultured for 48 h in a humidified chamber at 37 °C. The corresponding cancer cells were seeded into the upper compartments as a control. Similarly, to evaluate the macrophage features, Mφ cells were seeded into the lower compartment of the transwell, and the cancer cells or the corresponding Mφ cells were seeded into the upper compartment.

To evaluate the mechanism by which THP-1 macrophages regulate CD59 expression on PDAC cells, recombinant IL-6, neutralizing IL-6 antibody and the STAT3 pathway inhibitor AG490 were used. Recombinant IL-6 (Peprotech, Princeton, NJ, USA)) was dissolved in phosphate buffered saline (PBS) containing 5% trehalose (0.1 mg/ml), neutralizing IL-6 antibody (ab9324, Abcam) was dissolved in sterile water (0.1 mg/ml) and AG490 (Sigma-Aldrich, St Louis, MO, USA) was dissolved in DMSO (40 μmol/ml). These reagents were then diluted with the culture medium for experiments.

### siRNA transfection

Human CD59, IL-6 and STAT3 small-interfering RNAs (siRNAs) and control oligos were synthesized by RiboBio (Guangzhou, China). AsPC-1 and BxPC-3 cells were transfected with the siRNAs (50 nM) using Lipofectamine 3000 transfection reagent (Invitrogen, Carlsbad, CA, USA) according to the manufacturer’s protocol. The target sequences of the siRNAs used in this research were as follows: si-h-CD59 (5′-3′), GGTTACAAGTGTATAACAA; si-h-IL-6 (5′-3′), GGAGTTTGAGGTATACCTA; and si-h-STAT3 (5′-3′), CATCTGCCTAGATCGGCTA.

### Western blot analysis

We performed western blotting according to a standard protocol. Total cell lysates were extracted using 2% SDS lysis buffer with a protein phosphatase inhibitor mix (Applygen, Beijing), and 30 μg of total protein was prepared for electrophoresis through 8% or 12% (v/v) SDS–PAGE gels. After electrophoresis, the separated protein bands were transferred onto nitrocellulose membranes (Millipore, Ireland) and were blocked in 5% (w/v) Albumin Bovine-V (BSA-V, Solarbio, Beijing) for 1.5 h at room temperature. The primary antibodies used were as follows: anti-GAPDH (FL-335; Santa Cruz Biotechnology), anti-CD59 (HPA026494, Sigma-Aldrich), anti-CD163 (ab182422, Abcam), anti-arginase 1(Arg-1) (16001-1-AP, Proteintech), anti-IFN-γ (ab9657, Abcam), anti-iNOS (ab3523, Abcam), anti-IL-6 (ab6672, Abcam), anti-STAT3 (D1B2J, Cell Signaling Technology), and anti-phospho-Stat3 (Ser727, Cell Signaling Technology). The quality of loading and transferring was assessed by immunostaining with the anti-GAPDH antibody. The membranes were incubated with the primary antibodies at a dilution ratio of 1:1000 overnight at 4 °C. After washing three times in TBST, the membranes were incubated in 1:5000 HRP-conjugated secondary antibodies (Zsbio, Beijing) at room temperature for 1 h. Finally, the membranes were washed three times in TBST and visualized using an ECL Kit (Applygen, Beijing).

### Quantitative real-time PCR (qRT-PCR)

Quantitative real-time PCR was routinely performed, as previously described^19^. Total RNA was extracted from cells using TRIzol (Takara, Cat#9108) according to the manufacturer’s protocol. Complementary DNA (cDNA) was synthesized from total RNA using a reverse transcription kit (Takara, Cat. #RR037A) and quantitative real-time PCR was carried out using TB Green™ Premix Ex Taq™ (Tli RNaseH Plus) (Takara, Cat. #RR420A) according to the manufacturer’s instructions. β-actin was used as an internal control. The data were analyzed using the 2^−ΔΔCT^ method, and the output value of the control group was set to 1 automatically by the StepOnePlus system. The primer sequences used in this research were as follows: IL-6-F, AAAGAGGCACTGGCAGAAAA; IL-6-R, AGCTCTGGCTTGTTCCTCAC; CD163-F, TTTGTCAACTTGAGTCCCTTCAC; CD163-R, TCCCGCTACACTTGTTTTCAC; Arg-1-F, GGCTGGTCTGCTTGAGAAAC; Arg-1-R, ATTGCCAAACTGTGGTCTCC; IFN-γ-F, TGAATGTCCAACGCAAAGCA; IFN-γ-R, CTGGGATGCTCTTCGACCTC; iNOS-F, GCTCTACACCTCCAATGTGACC; iNOS-R, CTGCCGAGATTTGAGCCTCATG; β-actin-F, TGAAGGTAGTTTCGTGGATGC; and β-actin-R, TCCCTGGAGAAGAGCTACGA. Each experiment was performed in triplicate.

### Immunofluorescence staining

A total of 1.5 × 10^5^/ml pancreatic cancer cells were seeded into 6-well plates on 12-mm coverslips. After 24 h of culturing, the upper compartment of a transwell with a 0.4 μm pore size covered by 1.5 × 10^5^/ml pancreatic cancer cells or by the corresponding Mφ cells was put into the plates. After 48 h, the cells on the coverslips were fixed with 4% paraformaldehyde and were permeabilized with 0.1% saponin. Nonspecific staining was blocked by incubation with 5% goat serum/PBS for 1 h. Subsequently, the cells were incubated at room temperature for 1 h with the anti-CD59 antibody (HPA026494, Sigma-Aldrich) at a dilution of 1:200 and then incubated with a goat anti-rabbit Alexa Fluor 488 IgG (H + L) secondary antibody (Invitrogen, Cat # A-11034) for 1 h. Nuclei were strained with DAPI, and coverslips were placed face down onto a drop of antifading mounting medium on a microscope slide. Images were captured via a confocal laser scanning microscope (Nikon A1R) at 600X magnification. Each experiment was performed in triplicate.

### Flow cytometry analysis (FCM)

CD59 expression and CD163 expression on the cell membrane were detected by flow cytometry as described previously^20,21^. The single cell suspension was collected in ice-cold PBS and incubated with an FITC-conjugated anti-human CD59 antibody or PerCP-conjugated anti-human CD163 antibody (Biolegend, USA) in a darkroom for 40 min. Next, the stained cells were resuspended in PBS with 2% paraformaldehyde and were stored at 4 °C prior to flow cytometric analysis (Accuri C6, BD, USA). For each analysis, an isotype-matched monoclonal antibody was used as a negative control. Each experiment was carried out in triplicate.

### CDC and apoptosis assays

Pancreatic cancer cells were prepared as previously described. For the CDC assay, 2.0 × 10^5^/ml pancreatic cancer cells were seeded into 6-well plates, and the plates were incubated for 24 h at 37 °C and 5% CO_2_. Then, fresh human serum, diluted 2:5 in assay medium, or heat-inactivated serum was used as a control and was added and incubated for 24 h. Complement-mediated cell death of tumor cells was quantified by staining with annexin V/propidium iodide using an FITC Annexin V Apoptosis Detection Kit (Neobioscience, Shenzhen, China) according to the manufacturer’s instructions. All experiments were performed in triplicate.

### Enzyme-linked immunosorbent assay (ELISA)

The levels of IL-6 in the coculture media were determined using commercially available IL-6 ELISA kits by R&D Systems (Noves, USA) according to the manufacturers’ protocols. All samples were measured in triplicate with the provided immunoassay standard as a positive control. The ELISA plates were measured using a microplate reader at a wavelength of 450 nm; absorption was adjusted by subtracting background measurement results at 570 nm. A standard curve was created according to the manufacturers’ protocols and calculations were performed.

### RNA sequencing (RNA-seq)

Total RNA of AsPC-1 and THP-1 macrophage-treated AsPC-1 was freshly extracted and sequencing libraries were generated using the NEBNext UltraTM RNA Library Prep Kit for Illumina (NEB, USA) following the manufacturer’s protocol. RNA purity was verified using a NanoPhotometer spectrophotometer (IMPLEN, CA, USA) and the RNA integrity was assessed using the RNA Nano 6000 Assay Kit of the Bioanalyzer 2100 system (Agilent Technologies, CA, USA). Gene expression profiling and data analysis were conducted by the Beijing Novogene Experimental Department. All data were analyzed according to the manufacturer’s protocol. Differentially expressed genes were then identified according to fold change. The threshold set for up- and downregulated genes was a fold change greater than 2.

### Statistical analysis

The H-scores of CD59 staining and TAM infiltration in tumor and peritumor tissues were compared using the Mann–Whitney *U* test. IBM SPSS Statistics software version 21.0 and GraphPad Prism software version 5.0 were used for statistical analysis and for drawing the graphs. Overall survival was analyzed using the Kaplan-Meier product-limit method, and the significance of our variables was measured by the log-rank test. The Fisher exact test was used to analyze associations between two variables, and the Pearson Chi-square test was used to analyze associations between more than two variables. Multivariable analysis and analysis of continuous and ordinal variables was performed using the Cox proportional hazards regression method. A two-tailed *p*-value of *p* < 0.05 was significant.

## Results

### CD59 overexpression in pancreatic cancer tissues was associated with high histological grade and a poor prognosis of pancreatic cancer patients

**Fig. 1:**
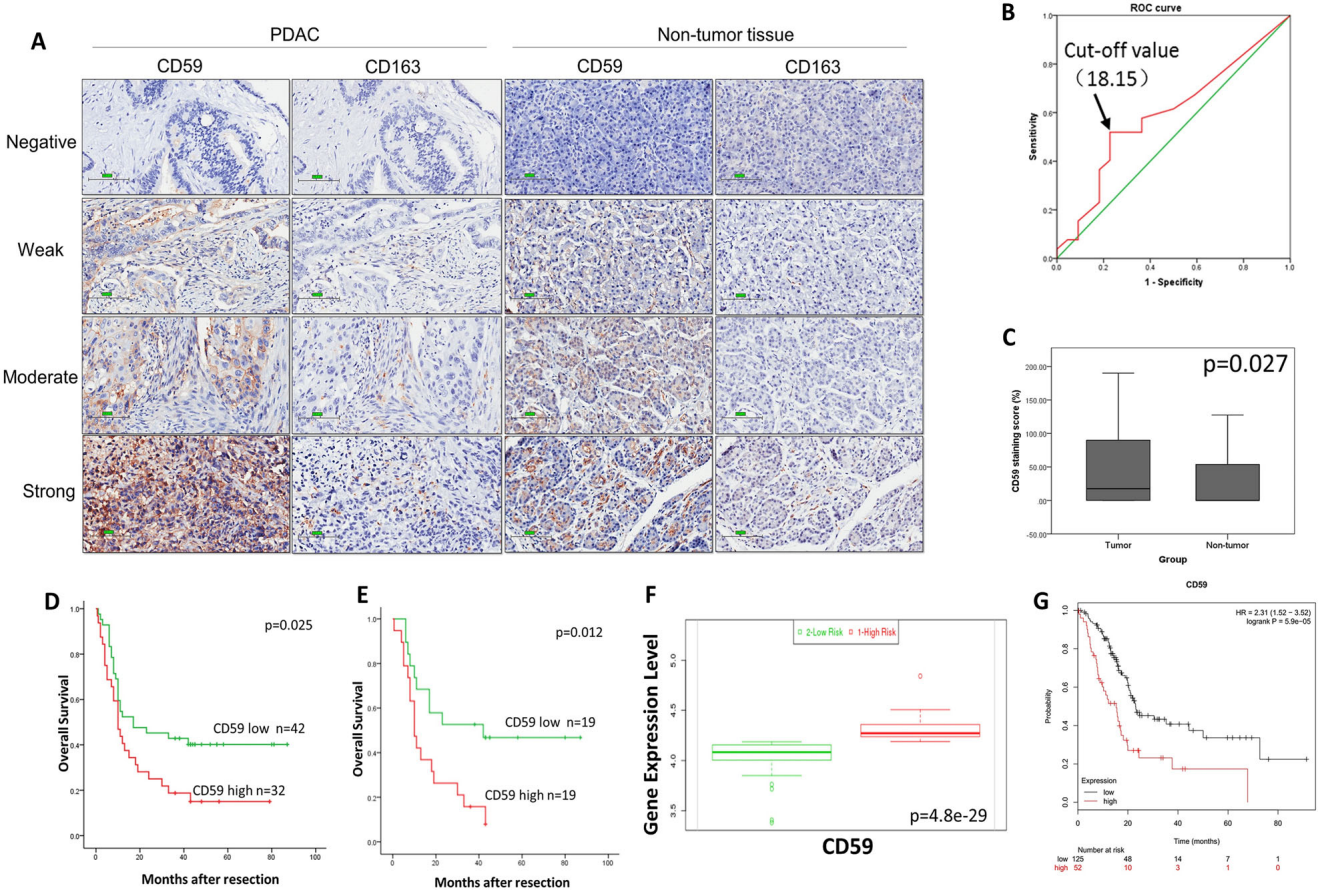
CD59 expression was upregulated in pancreatic cancer tissues. **a** Representative images of different levels of CD59 staining and CD163-positive TAM infiltration in tumor and non-tumor tissues by serial immunohistochemistry staining. Scale bar, 100 μm. **b** The ROC curve of CD59 for OS in pancreatic cancer tissues. **c** Comparison of the H-scores of CD59 between tumor and nontumor tissues (*p* = 0.027, Mann–Whitney *U* test). **d** The influences of tumoral CD59 expression on overall survival (*p* = 0.025, log-rank test). **e** Multivariate Cox regression analysis showed that CD59 expression was an independent prognostic marker in the age subgroup (age < 60 y, HR = 2.611, *p* = 0.012). **f** Comparison of CD59 expression between patients in the “High Risk” group and in the “Low Risk” group through the SurvExpress program (*p* < 0.001). **g** The prognostic value of CD59 mRNA expression in the Kaplan-Meier plotter dataset (HR = 2.31, *p* < 0.001)

CD59 expression levels as H-scores for pancreatic cancer tissues and adjacent nontumor tissues were different (Fig. 1a). Figure 1a shows the representative images of different staining intensities of CD59 in tumor tissues and the adjacent tissue. Based on cut-off values for clinicopathologic variables derived from the corresponding ROC curve (Fig. 1b), the median H-score of CD59 was 18.15 (range, 0–190). The H-score of CD59 was significantly higher in pancreatic cancer tissues than that in nontumor tissues (p = 0.027, Mann-Whitney *U* test, Fig. 1c). As shown in Table 1, the expression of CD59 in the tumor tissue was significantly associated with the histological grade (p = 0.034). No significant association was detected between CD59 expression and the other clinicopathological features. The effect of CD59 expression on the OS of the patients was detected using the Kaplan–Meier method and log-rank test. The univariate analysis showed that a worse overall patient survival was significantly associated with high CD59 expression in the tumor tissues (*p* = 0.025, Fig. 1d and Table 2), the N stage (*p* = 0.018) and the histological grade (*p* = 0.010) (Table 2). Multivariate Cox regression analysis showed that the histological grade and the N stage were independent prognostic markers (all *p* < 0.05) (Table 2) and that CD59 expression was an independent prognostic marker in the age subgroup (age < 60 y, HR = 2.611, *p* = 0.012, Fig. 1e and Table 3). Through the SurvExpress program, patients in the “High Risk” group presented a significantly higher expression level of CD59 than those in the “Low Risk” group (*p* < 0.001, Fig. 1f). According to the Kaplan–Meier plotter, the prognosis for the high-CD59 mRNA-expression level group was dramatically poorer than that for the lower level group (HR = 2.31, *p* < 0.001, Fig. 1g). Therefore, overexpression of CD59 may be a biomarker indicating worse survival for pancreatic cancer patients.

**Table 1:** Relationship between CD59 expression and clinicopathologic features of pancreatic cancer

Variables	Number	Tumoral CD59 expression		P value
		Low	High	
Age				
≤60 years	38	19	19	0.166
>60 years	36	23	13	
Gender				
Male	43	23	20	0.334
Female	31	19	12	
Tumor location				
Head	52	31	21	0.305
Body/tail	22	11	11	
T stage				
T1-2	47	24	23	0.144
T3	27	18	9	
N stage				
N0	44	25	19	0.589
N1	30	17	13	
Perineural invasion				
Negative	47	28	19	0.343
Positive	27	14	13	
Histological grade				
G1-2	49	32	17	**0.034**
G3	25	10	15

**Table 2:** Univariate and multivariate analyses of CD59 expression and clinicopathological parameters

Variables	Univariate analysis			Multivariate analysis		
	HR	95%CI	*P* value	HR	95%CI	*P* value
CD59 expression						
High vs Low	1.825	1.057–3.155	**0.025**	1.479	0.8509–2.570	**0.165**
Age (years)						
<60 vs ≥60	0.861	0.500–1.485	0.591			
Gender						
Male vs Female	0.959	0.553–1.663	0.881			
Tumor location						
Head vs Body/tail	1.093	0.612–1.954	0.763			
T stage						
T3 vs T1-2	1.417	0.786–2.557	0.244			
N stage						
N1 vs N0	1.946	1.121–3.378	**0.018**	2.996	1.590–5.646	**0.001**
Perineural invasion						
Positive vs Negative	1.342	0.772–2.336	0.297			
Histological grade						
G3 vs G1-2	2.083	1.193–3.650	**0.010**	3.020	1.582–5.765	**0.001**

**Table 3:** Multivariate Cox regression analyses of the prognostic relevance of CD59 expression in PDAC subgroups in which the poorer overall survival of patients was significantly associated with high CD59 expression

Subgroups	HR	95%CI	*P* value
Age (years) < 60	2.611	1.179–5.780	0.012

### TAM infiltration and CD59 expression were positively correlated in pancreatic cancer tissues

**Fig. 2:**
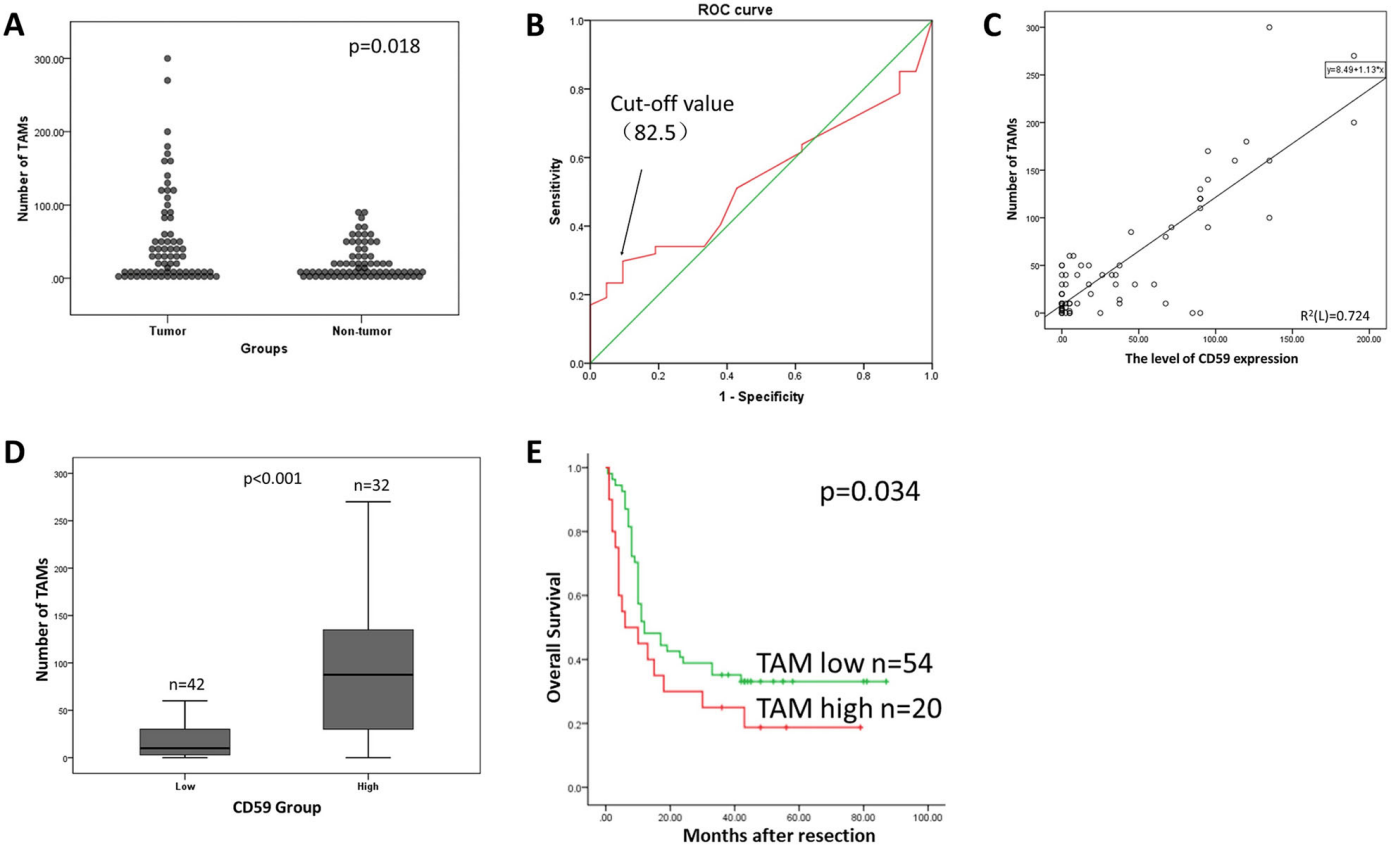
TAM infiltration and CD59 expression were positively correlated in pancreatic cancer tissues. **a** There was more TAM infiltration in tumor tissues than that in nontumor tissues (*p* = 0.018, Mann–Whitney *U* test). **b** The ROC curve of intratumoral TAMs for OS in pancreatic cancer tissues. **c** TAMs that infiltrated the tumor and CD59 expression were positively correlated (*R*^2^ = 0.724). **d** The number of TAMs that infiltrated the tumor tissues with high levels of CD59 expression was significantly higher than that in tissues with low CD59 expression (*p* < 0.001, Mann–Whitney *U* test). **e** The influence of tumoral TAM infiltration on OS (*p* = 0.034, log-rank test)

TAMs are a major constituent of the tumor immunosuppressive microenvironment and are known to stimulate key steps in tumor progression. Figure 1a shows representative images of serial section staining of CD163 and CD59 in tumor and adjacent nontumor tissues. The total number of intratumoral TAMs was significantly higher than that in nontumor tissues (*p* = 0.018, Mann–Whitney *U* test, Fig. 2a). The cut-off value of intratumoral TAMs was selected by the ROC curve as mentioned above, and the median number of TAMs was 82.5 (Fig. 2b). TAMs that infiltrated the tumor had a positive correlation with CD59 expression (*R*^2^ = 0.724, Fig. 2c). According to the classification of CD59 expression levels in tumor tissues, the number of TAMs that infiltrated the tumor tissues with a high level of CD59 expression was significantly higher than the number of TAMs in tissues with low CD59 expression (*p* < 0.001, Fig. 2d). Furthermore, worse OS was significantly associated with a high TAM infiltration in the tumor tissues using the Kaplan–Meier method and the log-rank test (*p* = 0.034, Fig. 2e). These data suggested that CD59 expression was proportionally correlated with TAM infiltration in pancreatic cancer tissues and that there might be cross-talk and cooperation between TAMs and CD59 expression in pancreatic cancer cells.

### THP-1 macrophages upregulated CD59 expression on cancer cells and protected cells from CDC in vitro

**Fig. 3:**
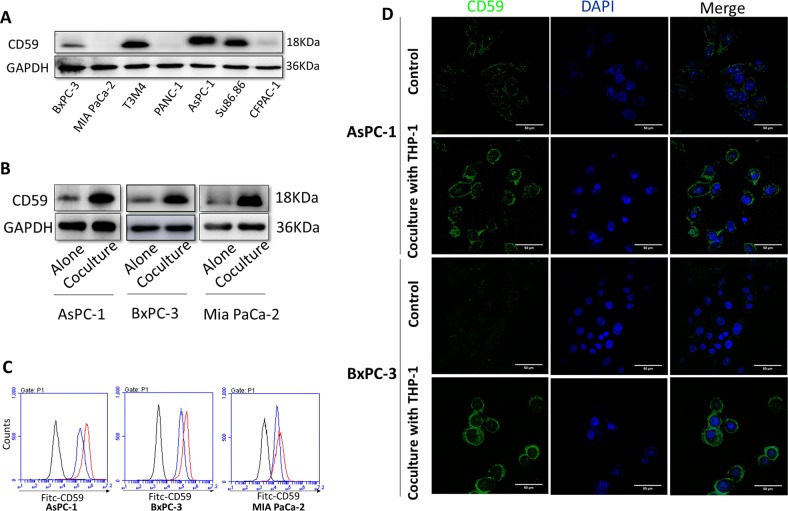
THP-1 macrophages upregulated CD59 expression on pancreatic cancer cells. **a** The expression levels of CD59 protein in seven pancreatic cancer cell lines. **b**, **c** CD59 expression of AsPC-1, BxPC-3, and MiaPaCa-2 cells was elevated in the group cocultured with THP-1 macrophages compared with expression in the control group detected by western blot and FCM. **d** The effects of macrophages on the CD59 expression of AsPC-1 and BxPC-3 were detected by immunofluorescence

**Fig. 4:**
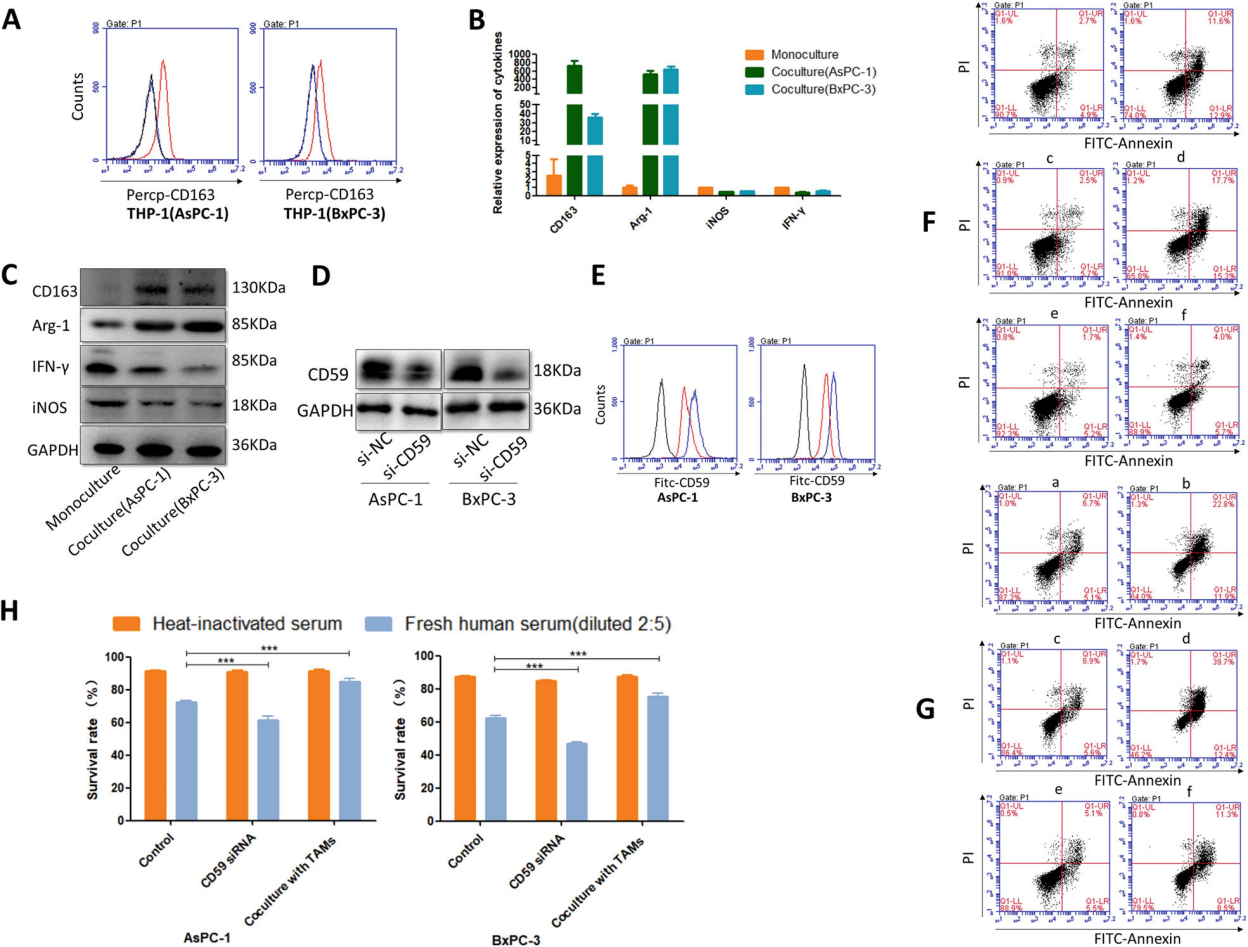
THP-1 macrophages protected cancer cells from CDC by overexpression of CD59. **a**–**c** The M2 phenotypes of the Mφ macrophages and the pancreatic cancer-cocultured macrophages were detected by FCM, qRT-PCR and western blot (**a**: the black line represents the negative control group, the blue line represents the monoculture group and the red line represents the THP-1 cocultured with AsPC-1 or BxPC-3; **b**, **c**: the monoculture: the Mφ macrophages, coculture: pancreatic cancer-cocultured macrophages). **d** CD59 siRNAs downregulated CD59 expression of AsPC-1 and BxPC-3 cells. **e** Flow cytometry analysis of CD59 on si-NC and si-CD59 cancer cells (the black line represents the negative control group; the blue line represents the si-NC group; and the red line represents the si-CD59 group). **f**, **g** CDC and apoptosis assay between the si-CD59 groups (AsPC-1: Fc, d and BxPC-3: Gc, d), the coculture groups (AsPC-1: Fe, f and BxPC-3: Ge, f) and the control groups (AsPC-1: Fa, b and BxPC-3: Ga, b). Fresh human serum, diluted 2:5 (AsPC-1: Fb, d, f and BxPC-3: Gb.d, f) in the assay mfedium, was used to provide complement components and heat-inactivated serum was used as the control (AsPC-1: Fa, c, e and BxPC-3: Ga, c, e). **h** The survival rate of THP-1 macrophage-cocultured cells markedly increased compared with that of the control group (left panel: AsPC-1 group, right panel: BxPC-3 group, ***p < 0.001)

To evaluate the effects of TAMs on CD59 expression in cancer cells, we examined the expression levels of CD59 protein in 7 human pancreatic cancer cell lines (BxPC-3, MiaPaCa-2, T3M4, PANC-1, AsPC-1, Su86.86, and CFPAC-1, Fig. 3a) and selected AsPC-1 as the high expression group. BxPC-3 and MiaPaCa-2 were selected as the medium and low expression groups for further study, respectively. We examined the effects of THP-1 macrophages on CD59 expression in these three cell lines by western blot and FCM. The CD59 expression in the three cell lines was elevated in the coculture group with THP-1 macrophages compared with expression in the control group (Fig. 3b, d). Since the CD59 expression level in MiaPaCa-2 was much lower than that in AsPC-1 and BxPC-3, the CD59 band of MiaPaCa-2 was almost invisible when detected together with the others (data not shown). Therefore, the western blots of the three groups were detected individually, and the results were clear (Fig. 3b). Therefore, AsPC-1 and BxPC-3 were chosen for additional experiments. The effect of TAMs on CD59 expression in these two cell lines was also confirmed by immunofluorescence staining (Fig. 3d). Activated THP-1 macrophages and pancreatic cancer cells were cocultured in the 0.4-μm transwell system, and the Mφ macrophages cocultured with pancreatic cancer cells became a CD163^+^ M2 phenotype (Fig. 4a), which was similar to the phenotype of TAMs in the tumor tissue. We also detected representative markers of THP-1 macrophages (CD163/Arg-1/IFN-γ/iNOS, Fig. 4b, c) induced by the pancreatic cancer cells at the mRNA and protein levels, according to the characteristics of TAMs. To dissect the function of CD59 in pancreatic cancer cells against CDC, we used siRNAs to inhibit CD59 expression in AsPC-1 and BxPC-3, which was detected by western blot and FCM (Fig. 4d, e). To determine whether the increase in CD59 expression by THP-1 macrophages could inhibit CDC, we conducted a CDC and apoptosis assay between the si-CD59, coculture and control groups. Then, we used fresh human serum, diluted 2:5, in the assay medium to provide the complement system and used heat-inactivated serum as the control. After reducing CD59 expression in pancreatic cancer cells, the survival rate of cells decreased in the presence of the complement system (Fig. 4f, g). After coculture with THP-1 macrophages, the survival rate of cells markedly increased compared with that of the control group (Fig. 4h, left panel: AsPC-1 group, right panel: BxPC-3 group, ***p < 0.001). Therefore, pancreatic cancer-educated macrophages could upregulate CD59 expression on cancer cells and protect cancer cells from CDC in an in vitro experiment.

### Pancreatic cancer-educated macrophages induced the upregulation of CD59 in pancreatic cancer cells via the IL-6R/STAT3 pathway

**Fig. 5:**
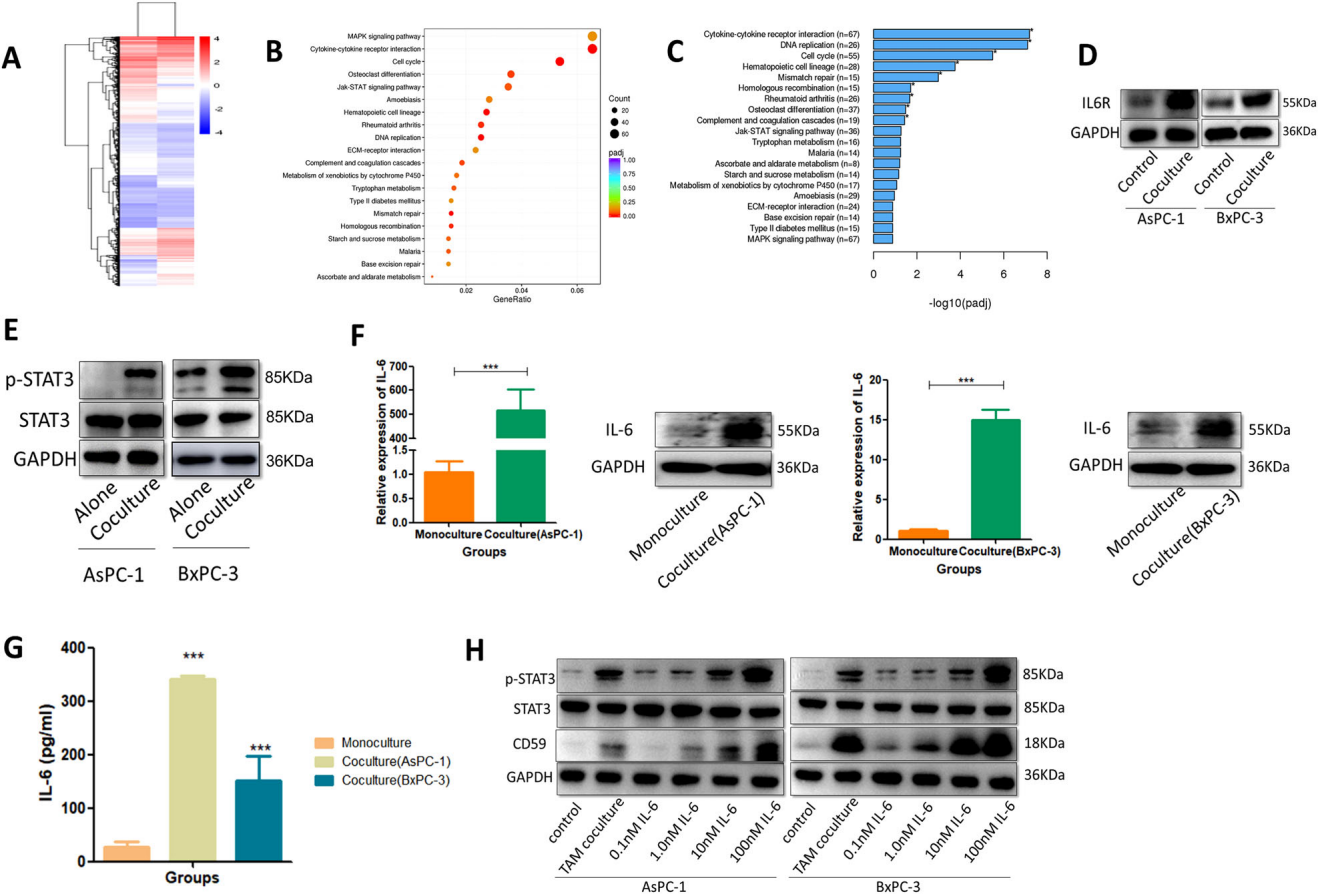
Pancreatic cancer-educated macrophages induced the upregulation of CD59 in cancer cells via the IL-6R/STAT3 pathway. **a** Gene expression profiling in AsPC-1 cells cocultured with THP-1 macrophages compared with AsPC-1 cells. **b**, **c** GO and KEGG pathway analyses of differentially expressed genes. **d** IL6-R was extremely elevated in the cocultured group compared with that in the control group. **e** AsPC-1 and BxPC-3 cells were cultured with THP-1 macrophages and analyzed for the level of total STAT3 or phosphorylated STAT3 (p-STAT3). **f**, **g** IL-6 expression in macrophages cocultured with AsPC-1 and BxPC-3 cells was detected by qRT-PCR, western blot and ELISA. **h** The CD59 expression and phosphorylation of STAT3 in pancreatic cancer cells incubated with various concentrations of recombinant IL-6 (0, 0.01, 1, 10, and 100 nM) were detected by western blot

**Fig. 6:**
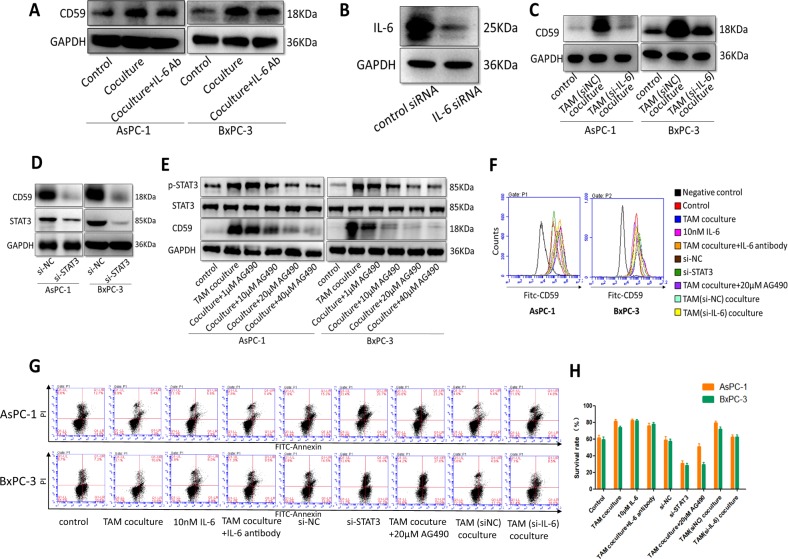
The effects on CD59 expression of IL6-antibody, si-IL-6, siSTAT3 and the STAT3 inhibitors. **a** IL-6 neutralization in the coculture system by IL-6 antibody inhibited CD59 expression in response to THP-1 coculture to a certain degree, but not significantly. **b** IL-6 siRNAs downregulated the IL-6 expression of THP-1. **c** After IL-6 knockdown of macrophages, CD59 upregulation was reversed in the coculture system. **d** siSTAT3 pancreatic cancer cells cocultured with THP-1 macrophages expressed lower amounts of CD59 than that of the siNC group. **e** STAT3 inhibitor, AG490 significantly decreased the expression of CD59 in THP-1-cocultured pancreatic cancer cells in a concentration-dependent manner. **f** The CD59 levels of the different representative groups were detected by FCM. **g**, **h** CDC and apoptosis assay between the representative groups in the conditioned media (fresh human serum, diluted 2:5)

Gene expression profiling was performed in AsPC-1 cocultured with THP-1 macrophages and in AsPC-1 alone (Fig. 5a). Gene Ontology (GO), Kyoto Encyclopedia of Genes and Genomes (KEGG) and Reactome pathway analyses of differentially expressed genes (*p* < 0.05) were performed (Fig. 5b, c). We found that the “cytokine-cytokine receptor interaction” category ranked 1st in the GO enrichment analysis out of all the differentially expressed genes; IL-6R was significantly elevated, and the result was confirmed by western blot (Fig. 5d). As mentioned before, CD59 expression on AsPC-1 and BxPC-3 was significantly elevated in the coculture group with macrophages compared with that in the control group, resulting in rapid phosphorylation of STAT3 (Fig. 5e). Considering that STAT3 is the major tumor-promoting effector of IL-6^22,23^, we focused on the STAT3 signal transduction pathway in promoting macrophage-mediated differentiation of pancreatic cancer cells. We hypothesized that IL-6, secreted by TAMs, might induce the upregulation of CD59 in pancreatic cancer cells via STAT3 activation. IL-6 expression of pancreatic cancer-educated macrophages was proven to be significantly increased using qRT-PCR and western blots (Fig. 5f), and IL-6 secreted by the macrophages cocultured with cancer cells increased markedly, as detected by ELISA (Fig. 5g). Then, the pancreatic cancer cells were incubated with various concentrations of IL-6 (0, 0.01, 1, 10 and 100 nM), and the CD59 expression and phosphorylation levels of STAT3 in cancer cells were significantly elevated to levels comparable to those in cells stimulated with THP-1 macrophages (Fig. 5h). To gain further insight into the importance of IL-6 in macrophages as a key upstream factor driving CD59 up-regulation in pancreatic cancer cells, we neutralized IL-6 from the supernatants of the coculture system via a commercially available blocking antibody. Neutralizing IL-6 within the coculture system led to a reduction in the ability of macrophages to upregulate CD59 expression in pancreatic cancer cells, but the result was not so remarkable (Fig. 6a). Then we performed the experiment to knockdown IL-6 by siRNA in the macrophages and used the macrophage conditioned media to stimulate cancer cells (Fig. 6b). We found that the knockdown of IL-6 in macrophages reduced CD59 expression much more so than the IL-6 blocking antibody (Fig. 6c). Subsequently, we transfected STAT3 siRNAs and siNC into pancreatic cancer cells. SiSTAT3 pancreatic cancer cells cocultured with THP-1 macrophages expressed lower amounts of CD59 than the siNC group (Fig. 6d). Then, we used AG490 to deplete STAT3 protein in pancreatic cancer cells as previously described^24^, and AG490 significantly decreased the expression of CD59 in cocultured pancreatic cancer cells in a concentration-dependent manner (Fig. 6e). The effects on CD59 expression in the representative condition were also detected by FCM (Fig. 6f). The CDC and apoptosis assays were performed in representative groups, and the protection conferred by CD59 was increased when recombinant IL-6 was used and reversed when IL-6 was knocked down (Fig. 6g, h). The survival rates in the si-STAT3 and AG490 groups were much lower, indicating that the STAT3 signaling pathway could affect the behavior of cancer cells in multiple ways. The effects of IL-6 antibody on CDC were not prominent, and this might be due to the degradation or low activity of antibody in the coculture media. These data demonstrated that M2 macrophages up-regulated CD59 expression in cancer cells via the IL-6R/STAT3 signaling pathway.

**Fig. 7:**
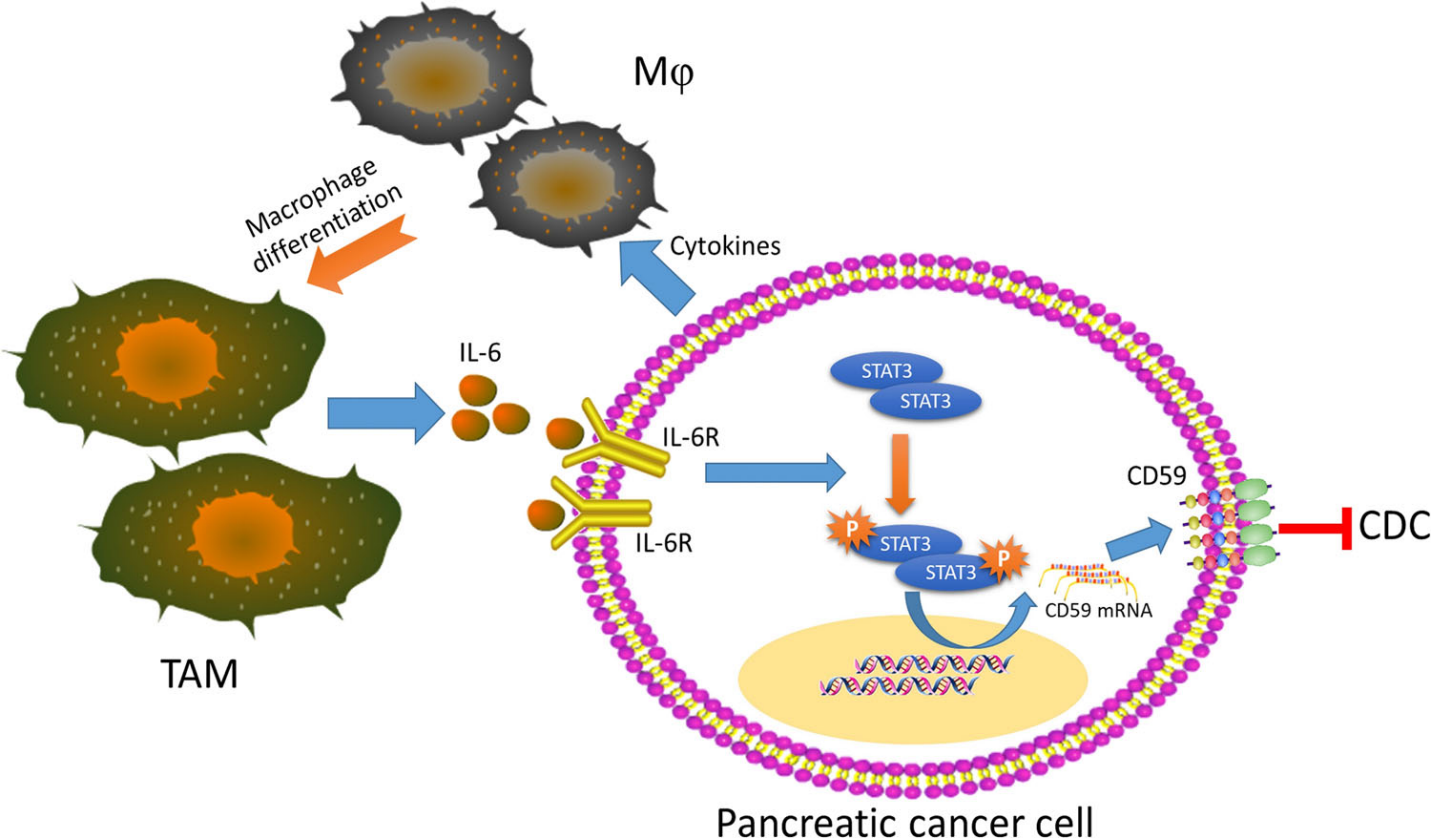
Schematic diagram of this study: TAMs protect pancreatic cancer from CDC by upregulating of CD59 in a paracrine IL-6R/STAT3 manner

In summary, these results revealed a novel interaction between TAMs and CD59 expression in pancreatic cancer cells, and demonstrated that pancreatic cancer-educated macrophages could protect pancreatic cancer cells from CDC by regulating CD59. These novel findings presented new therapeutic strategies of the immunotherapy of pancreatic cancer. (Fig. 7).

## Discussion

Pancreatic cancer is extremely malignant and its 5-year overall survival was <10%. The biomedical research on pancreatic cancer has shifted from the study of biological behavior and the regulation mechanisms of tumor cells themselves into the study of tumor microenvironments^25–27^. Cancer cells can be protected from CDC by high expression of CD59, and the function of immune cells in the tumor microenvironment can also be affected by CD59^8^. TAMs have been demonstrated to play an important role in tumor progression^28^. In this study, we showed that the expression levels of CD59 in clinical specimens were positively correlated with a worse prognosis for pancreatic cancer patients and that CD59 expression was positively correlated with TAM infiltration in pancreatic cancer tissues. We also demonstrated that IL-6 derived from pancreatic cancer-educated macrophages played vital roles in regulating the expression of CD59 in pancreatic cancer. This research explores new and promising therapeutic strategies for immunotherapy of pancreatic cancer.

High CD59 expression is generally associated with a worse prognosis in human cancers, including colorectal^29^, prostatic^30^, ovarian^31^, and lung^32^ cancers. Contradictory results have also been reported in several types of human cancers, such as breast cancer^33,34^. The role of CD59 in the progression and prognosis of pancreatic cancer has not yet been reported. Our study demonstrated that CD59 was overexpressed in pancreatic cancer tissues and that a high expression level was associated with a high histological grade and a worse prognosis. These results were also proven by online databases. CD59 expression in pancreatic cancer tissues was much higher than that in the surrounding tissues. Multivariate Cox regression analysis also showed that CD59 expression was an independent prognostic marker in the age subgroup (age < 60 y). It has been reported that CD59 inactivation or deficiency was associated with the development of atherosclerosis^35^ and the complications of diabetes^36^, and the incidence of these diseases increased significantly with age. This phenomenon may indicate that in elderly cancer patients, the benefits of low-levels of CD59 against cancer might counteract the effects of atherosclerosis or diabetes. However, it still requires further exploration. We also observed that CD59 expression was positively correlated with CD163^+^ M2 type macrophage infiltration in pancreatic cancer tissues. In the in vitro experiments, we found that pancreatic cancer cells were able to induce monocytes to differentiate into M2-type macrophages which could upregulate CD59 expression in cancer cells and protect cells from CDC. It is worth stating that the CD59 expression levels of different types of cancer cells vary. The change in CD59 expression after macrophage coculture is minimal for Mia PaCa-2 cells compared to AsPC-1 and BxPC-3 cells. Apart from CD59, CD46, and CD55 could also enable tumor cells to evade CDC and function as complement regulators. This may be due to MiaPaCa-2 cells mainly expressing CD46 or CD55 in addition to CD59, and future experiments should be performed. When the CDC assay was performed, the percentage of dead cancer cells was not as high as expected. This might be due to the protective function of CD46 or CD55 on cancer cells. Future experiments should be performed to investigate the effects of macrophages on CD46 or CD55 expression in cancer cells and the interactions between these complement restriction factors. Using gene expression profiling and bioinformatics analysis, we identified “cytokine-cytokine receptor interaction” as the main mechanism. Additional experiments have proven that IL-6 knockdown or STAT3 phosphorylation inhibition could reverse macrophage-induced CD59 upregulation. Here, we suggest that pancreatic cancer-educated macrophages induce the upregulation of CD59 in an IL-6R/STAT3-dependent manner in pancreatic cancer cells. In the tumor microenvironment, the IL-6R/STAT3 signaling pathway acted to promote tumor growth and progression^37^, and elevated circulating levels of IL-6 have been reported in patients with pancreatic^38^, breast^39^, colorectal^40^, and ovarian^41^ cancer types, among others. In this study, we observed that pancreatic cancer cells could induce macrophages to exhibit a tumor-promoting phenotype, and then the tumor-promoting macrophages induced CD59 expression in cancer cells by IL-6 secretion and by inducing IL-6R expression in cancer cells. IL-6 binds to IL-6R and results in the activation of STAT3, leading to the transcription of STAT3 target genes. This phenomenon will provide a novel mechanism of the TAM’s immunosuppressive function, which is to regulate the function of the complement systems. Although there are some studies on the complement components C5a and C1q regulating TAMs in recent years^42,43^, the mechanism of the regulation between TAMs and the complement system has not been thoroughly investigated. In our study, THP-1 was used as an in vitro model to investigate the function of macrophages. Future experiments should be performed with monocyte-derived macrophages or with TAMs isolated from PDAC patients to validate and confirm these findings with more cancer cells.

CD59 is highly expressed on cancer cells to regulate complement activation and is highly associated with chemotherapy resistance and radio resistance^8,44^. Therapeutic strategies that regulate the function of CD59 in the tumor immune microenvironment may be a promising method for tumor immunotherapy. Considering the wide distribution of CD59 on somatic cells, simply blocking the function of CD59 may induce potential side effects. Thus, a better understanding of the immunosuppressive networks regulating CD59 is needed. The IL-6/STAT3 pathway is hyperactivated in many types of cancers, and treatments that target the IL-6/STAT3 pathway have been widely studied and are listed as follows: directly targeting IL-6 with antibodies, such as siltuximab; targeting IL-6R with antibodies, such as tocilizumab; and targeting STAT3 inhibitors, such as peptidomimetics^37^. Targeting this signaling axis has been shown to be beneficial in the treatment of certain cancers. In this study, we demonstrated that TAMs induced the upregulation of CD59 in an IL-6R/STAT3-dependent manner and that IL-6 or STAT3 knockdown and the STAT3 pathway inhibitor AG490 could reverse the increase in CD59 expression caused by TAMs. Combined therapy by targeting TAMs, CD59 and IL-6 may be a direction for cancer treatments. Recent studies have shown that the potential application of bispecific and multispecific antibodies might provide new insights for cancer therapy. Evidence has shown that bispecific antibodies targeting CD59 and CD20 could increase the efficacy of immunotherapy in lymphocytic leukemia^20^. In recent studies, heterodimeric coiled coils were used as a tool to form polymers containing a variety of peptides^45,46^. Multispecific antibodies targeting TAMs, CD59, IL-6 and targetable cancer mutations might be a new promising strategy for cancer immunotherapy.

## Conclusions

In summary, crosstalk between macrophages and pancreatic cancer cells through the upregulation of CD59 in an IL-6R/STAT3 manner protects pancreatic cancer from CDC. This mechanism reveals promising insight into the exploration of novel therapeutic strategies for pancreatic cancer immunotherapy.
